# Bacterial Exposures and Associations with Atopy and Asthma in Children

**DOI:** 10.1371/journal.pone.0131594

**Published:** 2015-06-29

**Authors:** Maria Valkonen, Inge M. Wouters, Martin Täubel, Helena Rintala, Virissa Lenters, Ritva Vasara, Jon Genuneit, Charlotte Braun-Fahrländer, Renaud Piarroux, Erika von Mutius, Dick Heederik, Anne Hyvärinen

**Affiliations:** 1 Department of Health Protection, National Institute for Health and Welfare, Kuopio, Finland; 2 Department of Environmental Science, University of Eastern Finland, Department of Environmental Science, Kuopio, Finland; 3 Division of Environmental Epidemiology, Institute for Risk Assessment Sciences, Utrecht University, Utrecht, The Netherlands; 4 Institute of Epidemiology and Medical Biometry, Ulm University, Ulm, Germany; 5 University of Basel, Basel, Switzerland; 6 UMR MD3 Aix-Marseille University, Marseilles, France; 7 Ludwig Maximilians University, Munich, Germany; University of Naples Federico II, ITALY

## Abstract

**Background:**

The increase in prevalence of asthma and atopic diseases in Western countries has been linked to aspects of microbial exposure patterns of people. It remains unclear which microbial aspects contribute to the protective farm effect.

**Objective:**

The objective of this study was to identify bacterial groups associated with prevalence of asthma and atopy, and to quantify indoor exposure to some of these bacterial groups.

**Methods:**

A DNA fingerprinting technique, denaturing gradient gel electrophoresis (DGGE), was applied to mattress dust samples of farm children and control children in the context of the GABRIEL Advanced study. Associations between signals in DGGE and atopy, asthma and other allergic health outcomes were analyzed. Quantitative DNA based assays (qPCR) for four bacterial groups were applied on the dust samples to seek quantitative confirmation of associations indicated in DNA fingerprinting.

**Results:**

Several statistically significant associations between individual bacterial signals and also bacterial diversity in DGGE and health outcomes in children were observed. The majority of these associations showed inverse relationships with atopy, less so with asthma. Also, in a subsequent confirmation study using a quantitative method (qPCR), higher mattress levels of specifically targeted bacterial groups - *Mycobacterium* spp., *Bifidobacteriaceae* spp. and two different clusters of *Clostridium* spp. - were associated with a lower prevalence of atopy.

**Conclusion:**

DNA fingerprinting proved useful in identifying bacterial signals that were associated with atopy in particular. These findings were quantitatively confirmed for selected bacterial groups with a second method. High correlations between the different bacterial exposures impede a clear attribution of protective effects to one specific bacterial group. More diverse bacterial flora in mattress dust may link to microbial exposure patterns that protect against development of atopic diseases.

## Introduction

The prevalence of asthma and atopy has increased considerably over the last few decades in Western countries [1]. Development of these diseases seems more pronounced in children who live in cities compared to children who live on farms [2–3]. However, the causes of the lower risk for developing allergic diseases have been elucidated only partially and mechanisms that explain this protective ‘farm effect’ have not been unravelled in detail. There is evidence that exposure to microbes and microbial components may be responsible for the protective quality of growing up on a farm—by triggering the innate immune system and influencing T-cell regulation—but associations found for different microbial agents have not always been consistent across different studies [4–9]. It has been suggested that several independent microbial signals may play a role in activating the protective effects of farm environments [10]. More recently, diversity of microbial exposure has been associated with a reduced risk of asthma in population studies [11].

Here, we present a comprehensive approach combining qualitative analysis of bacterial profiles in house dust of children in rural areas with a second, quantitative methodology to confirm the initial findings. Specifically the confirmatory aspect of this work is advancement beyond previous studies on the relevance of microbial factors in asthma and allergy.

## Methods

### Samples and study design

The GABRIEL Advanced survey is an interdisciplinary study designed to identify the genetic and environmental causes of asthma in the European Community. The study design has been described previously [12] and is detailed in the supporting information. In brief, the main survey consisted of a weighted stratified random sample of a general population sample drawn from rural areas in Germany, Austria and Switzerland. Exposure strata were defined as (i) farm children, who lived on an operating farm; (ii) exposed non-farm children, who did not qualify as farm children but had regularly contact with farms and/or consumed farm milk; and (iii) unexposed non-farm children. For the current study, disproportionate random samples were drawn within each of the aforementioned exposure groups stratified by disease into asthmatic, non-asthmatic atopic, and non-asthmatic non-atopic children. Disproportionate sampling was used to increase statistical power for the analyses of the main characteristics under investigation: exposure to farming environments, asthma and atopy.

Mattress dust samples of 224 children aged 6–12 years were used in this analysis. General characteristics of the study population are described in Table 1. The dust samples were provided by the parents using a standardised dust collection protocol [13] which included photo instructions. The whole mattress was vacuumed for a period of 2 minutes, with a dust sampling nylon sock attached to the vacuum cleaner hose. The dust samples were stored at -80°C or below upon arrival at the study centre.

**Table 1:** General characteristics of the study population (n = 224).

	FARM CHILDREN			EXPOSED NON-FARM CHILDREN			NON-EXPOSED NON-FARM CHILDREN		
	ASTHMA	NO ASTHMA BUT ATOPY	NO ASTHMA OR ATOPY	ASTHMA	NO ASTHMA BUT ATOPY	NO ASTHMA OR ATOPY	ASTHMA	NO ASTHMA BUT ATOPY	NO ASTHMA OR ATOPY
N	32	23	19	32	22	21	32	21	22
Child sex (F/M)	10 / 22	10 / 13	13 / 6	12 / 20	12 / 10	6 / 15	11 / 21	5 / 16	9 / 13
Age mean (min-max)	9.5 (7.5–11.4)	9.6 (7.4–11.3)	9.6 (7.5–11.4)	9.6 (7.3–11.5)	9.5 (7.4–11.3)	9.5 (7.4–11.5)	9.2 (7.1–11.0)	9.3 (7.8–11.2)	9.3 (7.3–11.5)
No of siblings(0–1 sibling / more than 1 sibling) %	50 / 50	43.5 / 46.5	42.1 / 57.9	83.9 / 16.1	63.6 / 36.4	57.1 / 42.9	65.6 / 34.4	61.9 / 38.1	77.3 / 22.7
Parental smoking (no smoking / one or both parents are smoking) %	78.1 / 21.9	91.3 / 8.7	61.1 / 38.9	69.0 / 31.0	81.8 / 18.2	57.1 / 42.9	71.0 / 29.0	71.4 / 28.6	76.2 / 23.8

Written informed consent was obtained from the parents or guardians of all children participating in the study. Ethical approval was obtained from the ethics committees of the participating universities; Bavarian Medical Association, Ulm University, the cantons Luzern, Zurich and Thurgau, Medical University of Innsbruck and Medical University of Wroclaw. The data protection concept was approved by the regional data protection authorities (Baden-Württenberg) [12].

### Profiling bacterial communities

DNA was isolated from 20 mg accurately weighed (SBA 31, Scaltec) mattress dust with bead beating (Glass beads 212–300 μm, Sigma Aldrich). 2 × 10^6^ spores of *Geotrichum candidum* (UAMH 7863) strain were added to the samples as an internal control (see S1 Text for use of the internal control in calculation of qPCR results). Bead milling was followed by extraction using GenElute Plant kit (Sigma-Aldrich, Germany). Fingerprints of the bacterial communities were produced using target DNA amplification by PCR followed by DGGE [14]. Ultimately, each separate band in the generated, bacterial DNA fingerprints represents a distinct bacterium/bacterial group, so-called "bacterial taxonomic units". DNA bands were stained, visualised and the fingerprints were documented by photographing. The images were analysed with Bionumerics (version 4.61) software. A detailed description of the molecular biological analyses is provided in the supporting information (S1 Text).

The presence of individual bands in the mattress dust samples were associated to respiratory and allergic symptoms indicative of asthma and atopy, serum IgE levels and farm exposures. A complete list of the health and exposure variables used in the statistical analysis is provided in S1 Table. Three major health endpoints were analysed using several questions or data variables: asthma (6 variables); atopy (5 variables); and eczema (2 variables). In addition, 19 different variables defining farm-related exposures were used (see below details on statistical methods).

### Confirmation studies using quantitative PCR

Selected bands were isolated from the DNA fingerprints and subjected to a process resulting in obtaining sequence information of the respective bacterial groups, allowing for the identification of the bacterial signals (detailed in the supporting information, S1 Text). In order to obtain confirmation of associations between bacterial groups in the DNA fingerprints and health outcomes, we applied a second, quantitative approach. Quantitative PCR (qPCR) assays targeting bacterial groups of interest were optimized (S1 Text, S2 Table). qPCR analyses were applied to the original dust samples in order to obtain a quantitative measure for the following bacterial groups: *Mycobacterium* spp., *Bifidobacteriaceae* spp., *Clostridium* spp. cluster I and *Clostridium* spp. cluster XI. Associations of qPCR levels with doctor-diagnosed asthma and atopy were explored.

### Statistical methods

Associations between DGGE band density, and health and exposure variables (S1 Table) were analysed using Tobit regression to obtain an unbiased estimate of the average differences in band density given the many non-detects [15–16]. Density of the DGGE bands determined in Bionumerics software was used as a semi-quantitative measure, assuming differences in band densities represent relative differences in bacterial levels, as previously suggested to be the case for the DGGE method [14,17]. Adjustment was made for multiple comparisons by using the False Discovery Rate (FDR) [18]. All analyses were back-weighted to a general population sample [12]. Principal component analyses were performed for band-density to explore clustering of bands.

Bands with significant associations with doctor-diagnosed asthma or the broad phase 1 asthma definition, and/or atopy [defined as IgE antibodies against house dust mite, cat, and/or birch allergen (0.7 kU/L), or a positive reaction to the grass mix (0.35 kU/L)], and/or doctor-diagnosed atopic eczema were selected for band excision, cloning and sequencing (with the exception of one band, as explained in S1 Text). Four bands that showed several associations with environmental factors were also sequenced. Then, a limited set of bacterial targets was selected for confirmative qPCR analysis. This selection was based on 1) a protective associations of the DGGE band with asthma and/or atopy and/or eczema as defined above; 2) association with one or more of the environmental exposure variables of interest; and 3) successful band excision and sequence determination.

We assessed the exposure-response relationships between quantitative levels of selected bacterial groups determined via qPCR (log transformed), atopy [IgE against house dust mite, cat, and/or birch allergen (0.7 kU/L), or a positive reaction to the grass mix (0.35 kU/L)] and doctor-diagnosed asthma using survey weighted logistic-regression, fitted with a cubic B-splines term, and adjusted for sex. Shannon diversity index [19] was also explored by logistic regression and cubic splines. Modelling was performed using R version 3.0.0 (R Foundation for Statistical Computing, Vienna, Austria) using the survey package [20–21]. The optimal model was determined by minimizing the Akaike Information Criterion (AIC).

## Results

**Fig 1:**
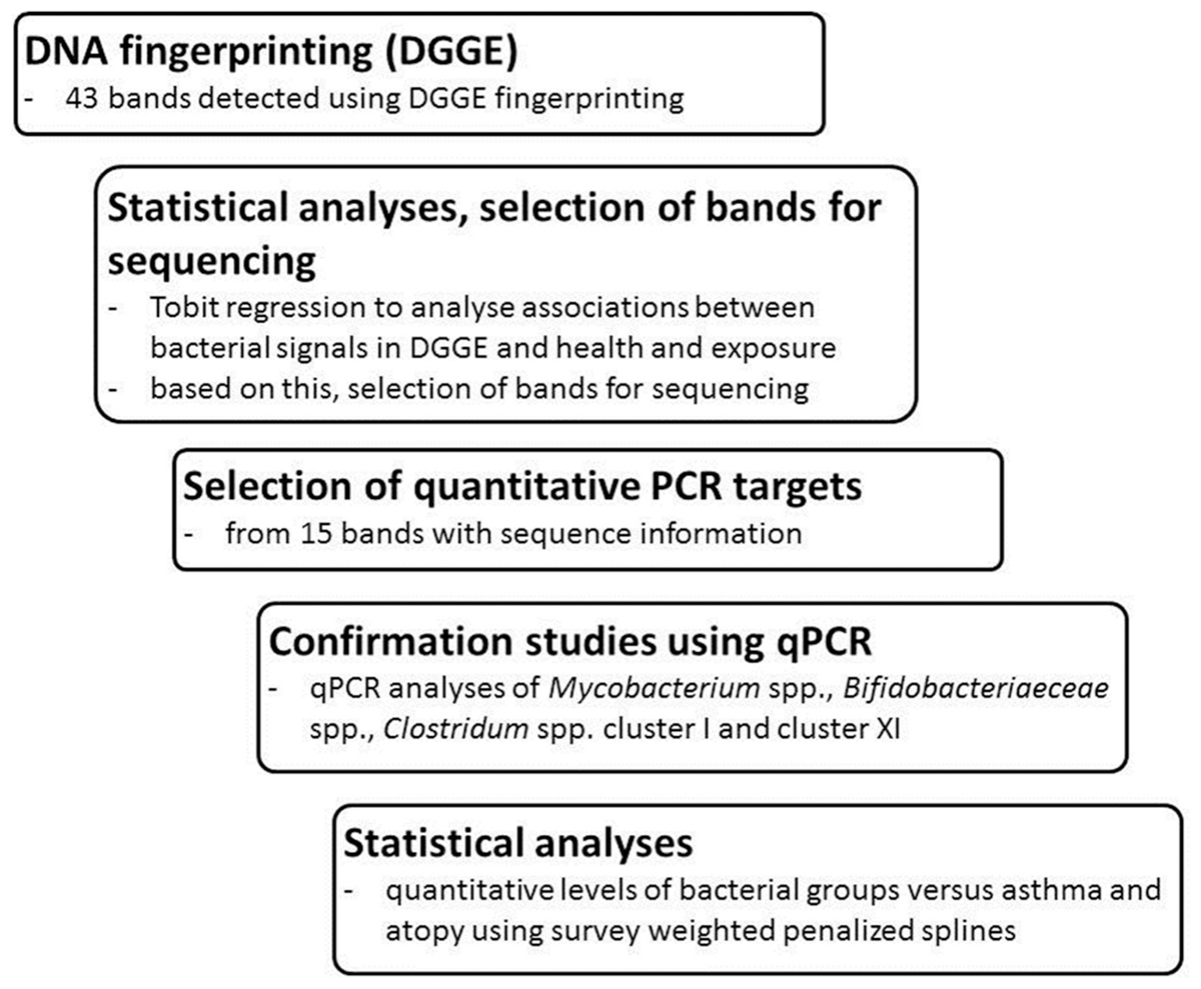
Flowchart of the study. Flowchart of molecular analyses (DGGE fingerprinting, quantitative PCR), statistical analyses, and the selection of bands for sequencing and qPCR assays.

The overall workflow of this study—including DNA fingerprinting of bacteria in house dust; statistical analyses of the DNA signals against health and exposure variables; selection of target signals for in depth studies (further sequencing); and quantitative confirmation of the results via qPCR—is illustrated in Fig 1.

### DNA fingerprinting

Altogether, 43 different bacterial bands representing distinct bacterial taxonomic units were found in the mattress dust DNA fingerprints. A median number of 14 bands per sample was found in 214 mattress dust samples with sufficient dust out of 224 samples. Of the 1376 explored relations, 142 had p-values <0.05. Of these 142, 33 associations remained statistically significantly associated with a health outcome or exposure variable after FDR adjustment. Most of the DGGE band associations with health outcomes were more frequently related to atopy and eczema than to asthma. One exception was band 22, which was strongly protectively associated with asthma and this association survived FDR adjustment. None of the associations with atopy (specific IgE cut point 0.7 kU/L) survived FDR adjustment; associations with hay fever and eczema symptoms survived FDR adjustment in four of the bands (band 29 and 39 associated with hay fever, band 20 and 38 associated with eczema symptoms).

### Selection of targets for sequencing

In total, 18 bands were subjected to cloning and DNA sequencing (Table 2). We obtained sequence information from 15 of these bands, out of which seven bands showed significant protective or risk associations with doctor diagnosed asthma, and/or atopy, and/or eczema. Sequence information retrieved for such bands pointed towards four distinct bacterial groups or taxonomic units and for those, qPCR assays were applied: *Mycobacterium* spp., *Bifidobacteriaceae* spp., and two different clusters of *Clostridium* spp. (cluster I and cluster XI). The bacterial taxonomic units targeted with qPCR assays were from distinctly different clusters (as tested in principal component analysis), indicating that the selected bands were not clearly statistically associated with each other.

**Table 2:** Overview of associations between the presence of 43 bacterial taxonomic units (= bands) identified with DNA fingerprinting in mattress dust, and their associations with the health outcomes asthma (6 variables), atopy (5 variables) and eczema (2 variables) and exposure variables (19 variables) (presented are the bands with an association with p<0.05 in Tobit regression analysis, adjusted for age and sex).

Band number	Health variables			Exposure variables			Selected for sequencing ¤
	Asthma (6 maximally) *	Atopy (5 maximally) **	Atopic eczema (2 maximally) ***	Farming activities ^#^	Consump-tion of farm milk †	Contact with animal feed ‡‡	
35			2 out of 2	x			y
			Protective				
41		3 out of 5		x		x	y
		Protective					
32		2 out of 5	1 out of 2	x	x		y
		Protective	Protective				
36		2 out of 5		x		x	y
		Protective					
17		2 out of 5		x			y
		Adverse Risk					
24			1 out of 2	x		x	y
			Adverse Risk				
8	1 out of 6			x	x		y
	Protective						
22	1 out of 6						y
	Protective						
20			1 out of 2				y
			Adverse Risk, 1 out of 2 Protective				
26			1 out of 2	x			y
			Protective				
37		1 out of 5			x		y
		Protective					
38		2 out of 5	1 out of 2	x		x	y
		Protective	Protective				
21	1 out of 6		1 out of 2				y
	Protective		Protective				
46	2 out of 6	2 out of 5		x	x	x	y
	Adverse Risk	Adverse Risk					
33				x			y
34				x	x		y
27				x		x	y
25				x	x	x	y
15			1 out of 2				
			Protective				
29		1 out of 5					
		Protective					
39		2 out of 5					
		Protective					
9	1 out of 6						
	Protective						
16	1 out of 6						
	Protective						
7				x			
14				x			
18				x	x		
23				x			
42				x			
44				x			
45				x		x	
47				x		x	
48				x		x	
11					x		
Nine bands without associations with atopy, asthma or any of the exposure variables							

* ‘yes’ to one or more (n/6) of the following six questionnaire items; broad phase 1 asthma definition [= reported wheeze (last 12 months or ever), asthma inhaler use ever, or a reported doctor’s diagnosis of asthma at least once, or wheezy bronchitis at least twice throughout the lifetime], doctor-diagnosed asthma, wheeze in the past 12 months, breathing problems and breathing noises, use of inhaler ever or in the past 12 months.

** ‘yes’ to one or more (n/5) of the following five items; specific IgE serum levels > 0.35kU/L, specific IgE serum levels > 0.7 kU/L, rhinitis symptoms in the past 12 months, rhinoconjunctivitis symptoms in the past 12 months, doctor diagnosed hay fever

*** ‘yes’ to one or two of the following: doctor diagnosed atopic eczema, eczema symptoms in the past 12 months

^#^ ‘yes’to one or more of the following items: child present during milking; cattle care; littering or removing dung; child spent time regular with animals in the stable

^†^ ‘yes’ to: has your child drunk milk directly from a farm regularly (at least once a week for 6 months)?

^‡‡^ ‘yes’ to: child present while the adults were feeding the animals

x = association (direction not defined) at least with one of the questions

y = yes, band selected to sequencing

^¤^ = Bands selected for sequencing had significant association with doctor diagnosed asthma or broad phase 1 asthma definition, and/or atopy (cut point 0.7kU/l), and/or atopic eczema. Bands 33, 34, 27, 25 were selected because of their multiple associations with farming factors.

Sequence information from bands with risk associations were related to the bacterial groups *Sphingomonas* spp. and *Paracoccus* spp. (Table 3).

**Table 3:** Detailed information on health associations of DGGE bands that were selected for sequencing and taxonomic allocation of sequences retrieved from the bands after database comparison (p-values of associations between DGGE bands and health outcomes are provided both after crude analyses and after FDR adjustment for multiple testing.

Band number		Associations with health outcomes	p-values	q-values (FDR adjusted)	Sequences retrieved from band with high (≥99%) database similarity	Likely taxonomic allocation
35	Protective	DD atopic eczema	0.008	p = 0.099	*Gardnerella* spp.	*Bifidobacteriaceae*
	Protective	Eczema symptoms	0.002	p = 0.083		
41	Protective	Specific IgE >0.7 kU/L	0.023		*Mycobacterium* spp., *Dietzia* spp.	*Corynebacterineae*
	Protective	Specific IgE >0.35kU/L	0.027			
	Protective	DD hay fever	0.038			
32	Protective	DD atopic eczema	0.006	p = 0.061	*Clostridium* spp.	*Clostridium*
36	Protective	Specific IgE >0.7 kU/L	0.003	p = 0.088		*Clostridiaceae*
	Protective	Specific IgE >0.35kU/L	0.002			
17	Risk	Specific IgE >0.7 kU/L	0.016		*Sphingomonadaceaea* spp.	*Sphingomonadaceae*
	Risk	Specific IgE >0.35kU/L	0.016			
24	Risk	DD atopic eczema	0.040		*Paracoccus* spp.	*Paracoccus*
8	Protective	DD asthma	0.025		No sequence information	
22	Protective	broad phase 1 asthma definition	0.008	p = 0.024	Ambiguous sequence information, no taxonomic allocation	
20	Risk	DD eczema	0.043	p = 0.001	No sequence information	
	Protective	eczema symptoms	0.000			
26	Protective	eczema symptoms (past 12 mo)	0.032		*Clostridiales* spp.	*Clostridiales*
37	Protective	rhinoconjunctivitis symptoms	0.040		*Psychrobacillus* spp.	*Bacillaceae*
38	Protective	Rhinoconjunctivitis symptoms	0.016	p = 0.005	*Rothia* spp.	*Micrococcaceae*
	Protective	DD hay fever	0.030			
	Protective	eczema symptoms	0.003			
21	Protective	inhaler use	0.040		*Corynebacterium* spp.	*Corynebacterium*
	Protective	eczema symptoms	0.035			
46	Risk	eczema symptoms	0.022	p = 0.080	No sequence information	
	Risk	rhinoconjunctivitis symptoms	0.024			
	Risk	DD hay fever	0.011			
33		No health associations			Ambiguous sequence information, no taxonomic allocation	
34		No health associations			*Actinomycetales* spp.	*Actinomycetales*
27		No health associations			*Sphingomonadaceae* spp.	*Sphingomonadaceae*
25		No health associations				*Enterobacteraceae*

Only q-values < 0.1are shown).

### Quantity and diversity of bacterial groups, relation to atopy and asthma

To confirm findings based on DNA-fingerprinting, the concentrations of four selected bacterial groups—*Mycobacterium* spp., *Bifidobacteriaceae* spp. and two different *Clostridium* spp. groups (cluster I and cluster XI)—were quantified via qPCR. The results presented as cells/mg dust are detailed for the nine exposure and health outcome strata in Table 4. The results are additionally expressed as microbial loads, i.e. cells per mattress area (cells/m^2^) in S3 Table. Loads of microbial markers expressed as cells per m^2^ were highly correlated (0.62–0.82, Spearman correlations); correlations for microbial concentrations (cells/mg) where somewhat lower (0.31–0.71, Spearman correlations).

**Table 4:** Levels of microbes detected using qPCR (cells/mg dust) and diversity metrics in three exposure strata stratified by health outcomes.

		FARM CHILDREN			EXPOSED NON-FARM CHILDREN			NON-EXPOSED NON-FARM CHILDREN		
		ASTHMA (N = 29)	NO ASTHMA BUT ATOPY (N = 23)	NO ASTHMA OR ATOPY (N = 19)	ASTHMA (N = 30)	NO ASTHMA BUT ATOPY (N = 22)	NO ASTHMA OR ATOPY (N = 18)	ASTHMA (N = 31)	NO ASTHMA BUT ATOPY (N = 20)	NO ASTHMA OR ATOPY (N = 22)
*Mycobacterium* spp.	Median	5573	6649	4927	3679	3989	4527	2284	2720	3343
	(min-max)	(1217–19031)	(2256–22142)	(1325–13239)	(573–12070)	(278–18519)	(559–9075)	(311–16741)	(121–6917)	(363–11967)
*Bifidobacteriaceae* spp.	Median	35603	24654	25252	17944	17703	20417	44919	14668	37983
	(min-max)	(1052–1.1x10E6)	(1315–286013)	(4051–7.2x10E6)	(2045–948785)	(1247–377892)	(1654–123586)	(3399–806926)	(1227–213290)	(543–566514)
*Clostridium* cluster I	Median	915	642	554	162	198	192	199	152	620
	(min-max)	(3–207616)	(23–36102)	(45–25647)	(6–4116)	(6–1472)	(11–540)	(9–1988)	(11–791)	(2–497132)
*Clostridium* cluster XI	Median	1931	1682	1308	343	243	225	340	183	409
	(min-max)	(28–99736)	(42–14942)	(92–40735)	(26–2769)	(2–2062)	(57–1645)	(18–3320)	(45–1415)	(18–3752)
Shannon diversity index	Median	2.68	2.65	2.68	2.56	2.62	2.71	2.59	2.54	2.68
	(min-max)	(1.89–3.09)	(2.24–2.91)	(2.34–2.88)	(1.93–2.95)	(0.68–3.01)	(2.30–3.05)	(2.36–2.88)	(2.01–3.02)	1.87–3.04)

**Fig 2:**
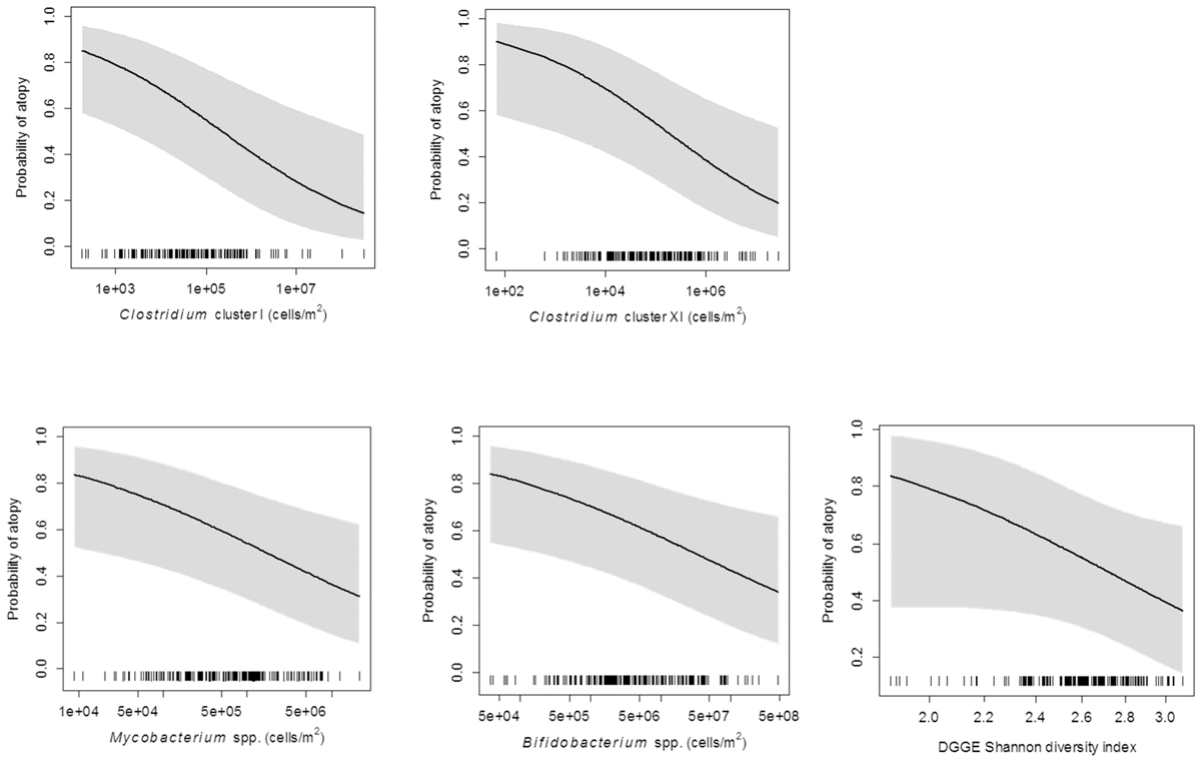
Logistic regression analyses for microbial determinations and atopy. Survey weighted logistic regression analyses fitted with a cubic spline term for qPCR results (in cells/m2 dust) in mattress dust and probability of atopy (0.7kU/L) in the whole study population. 95% confidence intervals displayed as shaded bands. One extremely small value (outlier) for the Shannon diversity index (0.68) was excluded.

Regression splines showed statistically significant inverse associations between qPCR-determined microbial levels in mattress dust and atopy (0.7 kU/L) (p-values = 0.004–0.019; Fig 2). There was also a protective trend (p-value = 0.097) of high bacterial diversity (Shannon index) on atopy (Fig 2). Models with a linear exposure term fit better (AIC) than models without an exposure term or with a exposure modelled with a cubic spline term. Individual bacterial groups and diversity were not clearly associated with asthma. Protective effects were clearly visible in the whole study population and in the strata of exposed and unexposed non-farm children (controls). Effects were generally weakest or non-existent within the farm children (data not shown).

## Discussion

We show here that levels of *Mycobacterium* spp., *Bifidobacteriaceae* spp. and particularly of *Clostridium* spp. cluster XI and I are inversely associated with atopy. These associations were seen in qualitative analyses using DGGE and were further confirmed when targeting these specific bacterial groups using qPCR. The confirmation of the indicative associations seen in DNA fingerprinting with a second, quantitative methodology is a major strength of our study and advances previous work on indoor microbial factors in asthma and allergy. The associations we observed were between distinct bacterial groups and atopy, which would suggest the possibility of an association between single microbial species and these diseases. However, associations between the levels of these bacterial groups and atopy were all significant and could not be mutually adjusted because of the high correlations.

The risk for atopy as a response to specific bacterial levels and diversity varied among the exposure groups. For children living on a farm, the protective effect of higher levels was weak or non-existent. Clearest effects of bacterial exposures were seen in the unexposed control population. Within this exposure stratum, the highest levels of *Clostridium* cluster I and XI and *Mycobacterium* spp. were seen in non-asthmatic, non-atopic subjects. Farm children are generally exposed to higher microbial levels throughout pregnancy and early childhood compared to non-farm children [4,22]. This more or less permanent, high-level exposure provides immune stimulus or challenge that probably protects from developing allergic disease later in life. This results in a population with high average microbial exposure levels, and lower prevalence of atopy. This might explain why an effect of high microbial levels within this sub-population might be more difficult to observe.

Levels of in particular *Clostridium* cluster XI and I, as well as *Mycobacterium* spp. were found to be highest in mattress dust from farm children. While *Clostridium* spp. are commonly found in the human intestinal tract, these bacteria are also present in other mammals and are abundant in milk products from farms. In particular *Clostridium* cluster I species are found in raw milk and milk products. Our findings suggest that these bacteria may represent exposures typically associated with farming environments. *Mycobacterium* spp. are less prevalent in humans or other animals, though some species are human and animal pathogens and endemically present in dairy cattle [23]. Sources of mycobacteria in house dust are manifold, including water, soil, aerosols and food. It is plausible that farming environments provide an additional rich source of this bacterial genus in house dust. *Bifidobacteriaceae* spp., on the other hand, occurred at comparably high levels in all exposure strata independent of farming environment contact, pointing towards the plausible relevance of the human source for this group of bacteria in mattress dust, though a clear source allocation cannot be provided here. *Bifidobacteriacea* spp. represent a major bacterial factor in the human intestinal flora and they are ubiquitous in the female vagina as well as the human oral flora. Nevertheless, members of this bacterial group is equally present in other mammals too. Interestingly, exposure to *Bifidobacterium* during the first year of life has—among other taxa determined from house dust in this respective study—been associated with less atopy in recent analyses of the Urban Environment and Childhood Asthma study in inner-city environments in several major cities in the United States [24].

The main objective of this study was to identify and quantitatively measure groups of bacteria which have associations with asthma and atopy and could possibly explain differences in prevalence of these diseases between rural and farm environments. Several recent papers [25–27] have synthesized the current knowledge on the farm effect and hygiene hypothesis; actual microbial factors underlying these phenomena have not been identified so far. While it is clear that exposure to microbes can influence the host immune system, protective effects of single microbial species in these diseases have been rarely explored. The findings in our study based on DNA fingerprinting of bacterial communities showed mostly inverse associations of individual bacterial signals with atopy and eczema, and much less so with asthma. Similarly, bacterial diversity in mattress dust in our study was protective for development of atopy, but not for asthma. This may for one partly be due to the limitation of small sample size. Ege et al. [11] reported inverse associations of fungal and bacterial diversity and specific microbial groups in house dust particularly with asthma, and only to a minor extent with atopy. It is relevant to explain here commonalities and differences between the earlier and this current study. Ege et al. presented results from two separate, complementary population studies: the PARSIFAL population consisted of children of farmers, children attending anthroposophic schools, and their respective reference groups living in rural and suburban areas of Bavaria. In the PARSIFAL study, Ege et al. focused on bacteria—as we did in our current study—and the main findings in PARSIFAL were that bacterial diversity and individual bacterial groups were inversely related to asthma, while findings were not significant for atopy. Differences in the study populations and in the molecular techniques applied may explain differences in the findings of PARSIFAL and our current study. The second population presented [11] were 450 subjects from the GABRIEL study population, which is overlapping with the one in our current analysis. However, the sample materials and especially the microbial determination methods differed fundamentally between the earlier and our current study. The earlier paper presented results on *viable* fungi and bacteria determined from airborne settled dust collected with an electrostatic dust collector. Inverse association of viable fungal diversity and specific fungal genera with asthma were highlighted, contrary to the bacterial focus in mattress dust in our current study. With respect to atopy, the presence of gram negative rods explained 30% of the effect of the farming environment on atopy. Our current study analysed specifically bacterial communities in mattress dust in a smaller sub-population of 214 study subjects of the GABRIEL survey. Here, we use DNA-based methodology that is not restricted to detection of viable cells only and targeted bacterial communities specifically. The differences in how and which aspects of the microbial exposures were assessed helps to explain why our study found more consistent associations of bacterial community factors with atopy, while the earlier study found associations of fungal groups and diversity with asthma. Our study presents quantitative confirmation of the qualitative findings from DNA fingerprinting, which has not been attempted in earlier studies. For this confirmation study we limited to targeting bacterial signals that had shown protective associations with atopy and eczema in DGGE.

While diversity within the human microbiome has been linked to several diseases [28], a possible impact of diversity in environmental microbial exposure on human health is much less explored. More diverse microbial exposure encountered in farm environments might have a protective quality in the development of allergic health outcomes [10–11]. The results of our study and earlier work on biodiversity and its interrelation with allergy support this hypothesis [29]. Improved measures of environmental microbial diversity will be provided by studies using next generation sequencing approaches that offer resolution of microbial communities at a very high level. It will be the subject of future research to investigate whether or not microbial diversity as such can explain beneficial effects on human health, or rather is a surrogate for a beneficial exposure situation.

We recognize certain limitations in the methodology of our study. Mattress dust is a convenient sample material in large epidemiological studies, but may not be the best sample to represent human exposure to environmental microbes. While bacterial content of mattress dust in urban homes is largely dominated by human-derived bacteria [30], this sample type, however, also reflects exposure to environmental microbes, for example farm contact [31]. A methodological limitation of virtually all DNA-based approaches that are currently being applied is that sequence fragments are limited in length, which typically prevents species level identification of microbial signals of interest. Since the rise of next generation sequencing approaches that now start to be used also in larger epidemiological studies on indoor microbial factors, the DNA fingerprinting method applied here may seem outdated, specifically due to limitations in resolution. However, the limitation that only the more abundant taxa of the microbial content and diversity in a sample are displayed in DGGE should equally be considered a strength, as these more abundant taxa likely also represent the more relevant groups from an exposure point of view. Increasing sequencing depth in next generation sequencing approaches is powerful, but also adds more of the less abundant and possibly not very etiologically-relevant bacterial taxa to the dataset, complicating a meaningful statistical analyses and identification of individual, relevant exposures. Despite this, there is little doubt that next generation sequencing approaches will be of great help in answering relevant questions on health effects of environmental microbial exposures.

In conclusion, this study adds to the body of evidence on protective qualities of microbial exposure in house dust, in particular observed for atopy. We succeeded in identifying bacterial signals that were more commonly found in mattress dust of non-atopic children. Within this study, we were able to confirm and specify these findings by quantifying the exposure to identified bacterial groups, which suggested protective associations for atopy, but not for asthma. High levels for *Clostridium* cluster I and XI and *Mycobacterium* spp. were seen in farming homes compared to non-farming homes. These findings suggest that these bacterial groups may contribute to a farm effect, offering protection from allergic health outcomes.

## Supporting Information

S1 Table List of the questions about health outcomes and environmental factors and clinical data (for atopy definition) analysed against DGGE bands. (DOC)

S2 Table QPCR assays applied on mattress dust samples in this study: target group, oligonucleotide sequence, optimized run parameters and reference to the original publications of the assays. (DOC)

S3 Table Levels of microbes detected using qPCR (cells/m^2^ dust) in the three exposure strata stratified by health outcomes. (DOC)

S1 Text Detailed information about DGGE, cloning sequencing and qPCR. (DOC)

## References

[1] James AL, Knuiman MW, Divitini ML, Hui J, Hunter M, Palmer LJ, et al Changes in the prevalence of asthma in adults since 1966: The Busselton health study. Eur Respir J 2010; 35(2): 273–278. 10.1183/09031936.00194308 19643935

[2] von Mutius E, Vercelli D. Farm living: effects on childhood asthma and allergy. Nat Rev Immunol 2010; 10(12): 861–868. 10.1038/nri2871 21060319

[3] Eder W, Ege MJ, von Mutius E. Current concepts: The asthma epidemic. N Engl J Med 2006; 355(21): 2226–2235. 17124020 10.1056/NEJMra054308

[4] Braun-Fahrlander C, Riedler J, Herz U, Eder W, Waser M, Grize L, et al Environmental exposure to endotoxin and its relation to asthma in school-age children. N Engl J Med 2002; 347(12): 869–877. 12239255 10.1056/NEJMoa020057

[5] Riedler J, Braun-Fahrländer C, Eder W, Schreuer M, Waser M, Maisch S, et al Exposure to farming in early life and development of asthma and allergy: a cross-sectional survey. Lancet 2001; 358(9288):1129 11597666 10.1016/S0140-6736(01)06252-3

[6] Sordillo JE, Hoffman EB, Celedón JC, Litonjua AA, Milton DK, Gold DR. Multiple microbial exposures in the home may protect against asthma or allergy in childhood. Clinical & Experimental Allergy 2010; 40(6): 902–910. 20412140 10.1111/j.1365-2222.2010.03509.x PMC3730840

[7] Chrischilles E, Ahrens R, Kuehl A, Kelly K, Thorne P, Burmeister L, et al Asthma prevalence and morbidity among rural Iowa schoolchildren. J Allergy Clin Immunol 2004; 113(1): 66–71. 14713909 10.1016/j.jaci.2003.09.037

[8] Ege MJ, Frei R, Bieli C, Schram-Bijkerk D, Waser M, Benz MR, et al Not all farming environments protect against the development of asthma and wheeze in children. J Allergy Clin Immunol 2007; 119(5): 1140–1147. 17349684 10.1016/j.jaci.2007.01.037

[9] Merchant JA, Naleway AL, Svendsen ER, Kelly KM, Burmeister LF, Stromquist AM, et al Asthma and farm exposures in a cohort of rural Iowa children. Environ Health Perspect 2005; 113(3): 350–356. 15743727 10.1289/ehp.7240 PMC1253764

[10] Ege MJ, Mayer M, Schwaiger K, Mattes J, Pershagen G, van Hage M, et al Environmental bacteria and childhood asthma. Allergy 2012; 67(12): 1565–71 10.1111/all.12028 22994424

[11] Ege MJ, Mayer M, Normand AC, Genuneit J, Cookson WO, Braun-Fahrlander C, et al Exposure to environmental microorganisms and childhood asthma. N Engl J Med 2011; 364(8): 701–709. 10.1056/NEJMoa1007302 21345099

[12] Genuneit J, Buchele G, Waser M, Kovacs K, Debinska A, Boznanski A, et al The GABRIEL Advanced Surveys: study design, participation and evaluation of bias. Paediatr Perinat Epidemiol 2011; 25(5): 436–447. 10.1111/j.1365-3016.2011.01223.x 21819425

[13] Schram-Bijkerk D, Doekes G, Douwes J, Boeve M, Riedler J, Ublagger E, et al Bacterial and fungal agents in house dust and wheeze in children: the PARSIFAL study. Clin Exp Allergy 2005; 35(10): 1272–1278. 16238785 10.1111/j.1365-2222.2005.02339.x

[14] Muyzer G, de Waal EC, Uitterlinden AG. Profiling of complex microbial populations by denaturing gradient gel electrophoresis analysis of polymerase chain reaction-amplified genes coding for 16S rRNA. Appl Environ Microbiol 1993; 59(3): 695–700. 7683183 10.1128/aem.59.3.695-700.1993 PMC202176

[15] Lotz A, Kendzia B, Gawrych K, Lehnert M, Brüning T, Pesch B. Statistical methods for the analysis of left-censored variables. GMS Medizinische Informatik, Biometrie und Epidemiologie 2013; 9(2).

[16] Lubin JH, Colt JS, Camann D, Davis S, Cerhan JR, Severson RK, et al Epidemiologic evaluation of measurement data in the presence of detection limits. Environ Health Perspect 2004; 112(17): 1691–1696. 15579415 10.1289/ehp.7199 PMC1253661

[17] Dilly O, Bloem J, Vos A, Munch JC. Bacterial diversity in agricultural soils during litter decomposition. Appl Environ Microbiol 2004; 70(1): 468–474. 14711676 10.1128/AEM.70.1.468-474.2004 PMC321295

[18] Benjamini Y, Hochberg Y. Controlling the false discovery rate: a practical and powerful approach to multiple testing. Journal of the Royal Statistical Society 1995, Series B 57 (1): 289–300. MR 1325392.

[19] Boon N, Windt W, Verstraete W, Top EM. Evaluation of nested PCR-DGGE (denaturing gradient gel electrophoresis) with group-specific 16S rRNA primers for the analysis of bacterial communities from different wastewater treatment plants. FEMS Microbiol Ecol 2002; 39(2): 101–112. 10.1111/j.1574-6941.2002.tb00911.x 19709189

[20] Lumley T. Survey: Analysis of complex survey samples”. R package version 3 22–1 2012.

[21] Lumley T. Analysis of complex survey samples. Journal of Statistical Software 2004; 9(1): 1–19.

[22] Karkkainen PM, Valkonen M, Hyvarinen A, Nevalainen A, Rintala H. Determination of bacterial load in house dust using qPCR, chemical markers and culture. J Environ Monit 2010; 12(3): 759–768. 10.1039/b917937b 20445866

[23] Eisenberg SW, Nielen M, Santema W, Houwers DJ, Heederik D, Koets AP. Detection of spatial and temporal spread of Mycobacterium avium subsp. paratuberculosis in the environment of a cattle farm through bio-aerosols. Vet Microbiol 2010; 143(2–4): 284–292. 10.1016/j.vetmic.2009.11.033 20036081

[24] Lynch SV, Wood RA, Boushey H, Bacharier LB, Bloomberg GR, Kattan M, et al Effects of early-life exposure to allergens and bacteria on recurrent wheeze and atopy in urban children. J Allergy Clin Immunol 2014; 134(3): 593–601 10.1016/j.jaci.2014.04.018 24908147 PMC4151305

[25] Frei R, Lauener RP, Crameri R, O'Mahony L. Microbiota and dietary interactions: an update to the hygiene hypothesis? Allergy 2012; 67(4): 451–461. 10.1111/j.1398-9995.2011.02783.x 22257145

[26] Wlasiuk G, Vercelli D. The farm effect, or: when, what and how a farming environment protects from asthma and allergic disease. Curr Opin Allergy Clin Immunol 2012; 12(5): 461–466. 10.1097/ACI.0b013e328357a3bc 22892709

[27] Heederik D, von Mutius E. Does diversity of environmental microbial exposure matter for the occurrence of allergy and asthma? J Allergy Clin Immunol. 2012; 130(1): 44–50. 10.1016/j.jaci.2012.01.067 22502794

[28] Human Microbiome Project Consortium. Structure, function and diversity of the healthy human microbiome. Nature 2012 13; 486(7402): 207–214. 10.1038/nature11234 22699609 PMC3564958

[29] Hanski I, von Hertzen L, Fyhrquist N, Koskinen K, Torppa K, Laatikainen T, et al Environmental biodiversity, human microbiota, and allergy are interrelated. Proc Natl Acad Sci USA 2012 109(21): 8334–8339 10.1073/pnas.1205624109 22566627 PMC3361383

[30] Taubel M, Rintala H, Pitkaranta M, Paulin L, Laitinen S, Pekkanen J, et al The occupant as a source of house dust bacteria. J Allergy Clin Immunol 2009; 124(4): 834–40 10.1016/j.jaci.2009.07.045 19767077

[31] Waser M, Schierl R, von Mutius E, Maisch S, Carr D, Riedler J, et al Determinants of endotoxin levels in living environments of farmers' children and their peers from rural areas. Clin Exp Allergy 2004; 34(3): 389–397. 15005732 10.1111/j.1365-2222.2004.01873.x

