# Supplementary material for: Bacterial Exposures and Associations with Atopy and Asthma in Children
Source: PLoS One. 2015 Jun 29;10(6):e0131594. doi: 10.1371/journal.pone.0131594 (PMC4488145; doi:10.1371/journal.pone.0131594)
Supplement: S1 Table — (DOC) [file pone.0131594.s001.doc]

S1 Table. List of the questions about health outcomes and environmental factors and clinical data (for atopy definition) analysed against DGGE bands.

| **Health outcomes** |  |
| --- | --- |
| **Asthma** |  |
| 1 | Broad phase 1 asthma definition: reported wheeze (last 12 months or ever), asthma inhaler use ever, or a reported doctor’s diagnosis of asthma at least once, or wheezy bronchitis at least twice throughout the lifetime |
| 2 | report of doctor diagnosed asthma |
| 3 | wheeze in the last 12 months |
| 4 | breathing problems and breathing noises |
| 5 | use of inhaler in the last 12 months |
| 6 | use of inhaler ever |
| **Atopy** |  |
| 7/8 | specific IgE serum levels >0.35 kU/L / >0.7 kU/L |
| 9 | doctor diagnosis of hay fever ever |
| 10 | rhinitis symptoms in the last 12 months |
| 11 | rhinoconjunctivitis symptoms in the last 12 months |
| **Eczema** |  |
| 12 | doctor diagnosis of atopic eczema ever |
| 13 | eczema symptoms in the past 12 months |
|  |  |
| **Environmental factors** | |
| **Consumption of farm milk** |  |
| 14 | child regularly consumed farm milk (at least once a week over a period of 6 months) |
| **Farming activities**  **(up to the age of 3 yrs)** |  |
| 15/16 | joined parents when milking cows (at least once a month/once a week) |
| 17/18 | joined parents when littering cow stable (at least once a month/once a week) |
| 19/20 | joined parents when performing cattle care (at least once a month/ once a week) |
| 21/22 | stayed in cow stable independent of parents (at least once a month/once a week) |
| 23/24 | at least one of 15 or 17 or 19 or 21 (at least once a month/once a week) |
| 25/26 | stayed in barn (at least once a month/once a week) |
| 27 | stayed on a farm |
| 28 | child has regular contact to a barn |
| 29 | child has regular contact to a stable |
| 30 | child lives on a farm |
| 31/32 | joined parents when feeding cows (at least once a month/once a week) |
