# Supplementary material for: Bacterial Exposures and Associations with Atopy and Asthma in Children
Source: PLoS One. 2015 Jun 29;10(6):e0131594. doi: 10.1371/journal.pone.0131594 (PMC4488145; doi:10.1371/journal.pone.0131594)
Supplement: S2 Table — (DOC) [file pone.0131594.s002.doc]

S2 Table. qPCR assays applied on mattress dust samples in this study: target group, oligonucleotide sequence, optimized run parameters and reference to the original publications of the assays.

| qPCR assay | Oligonucleotide sequence (5´- 3´) ^a^ | Cycling parameters | Published |
| --- | --- | --- | --- |
| *Mycobacterium*  spp. | F: GATGCAACGCGAAGAACCTT | 15 min at 95 °C,  40 cycles of 15 s at 95 °C, 60 s at 60 °C | Torvinen et al. 2010 (3) |
|  | R: TGCACCACCTGCACACAGG |  |  |
|  | P: FAM-CCTGGGTTTGACATGCACAGGACG-TAMRA |  |  |
| *Bifidobacteriacea*  spp. | F: GCGTGCTTAACACATGCAAGTC | 15 min at 95 °C,  40 cycles of 15 s at 95 °C, 60 s at 60 °C | Penders et al. 2005  (6) |
|  | R: CACCCGTTTCCAGGAGCTATT |  |  |
|  | P: FAM-TCACGCATTACTCACCCGTTCGCC-BHQ1 |  |  |
| *Clostridium*  cluster I | F: TACCHRAGGAGGAAGCCAC | 15 min at 95 °C,  40 cycles of 15 s at 95 °C, 60 s at 62 °C | Song et al. 2004  (4) |
|  | R: GTTCTTCCTAATCTCTACGCAT |  |  |
|  | P: FAM-GTGCCAGCAGCCGCGGTAATACG-BHQ1 |  |  |
| *Clostridium*  cluster XI | F: ACGCTACTTGAGGAGGA | 15 min at 95 °C,  40 cycles of 15 s at 95 °C, 60 s at 62 °C | Song et al. 2004 |
|  | R: GAGCCGTAGCCTTTCACT |  |  |
|  | P: FAM-GTGCCAGCAGCCGCGGTAATACG-BHQ1 |  |  |

^a^ F forward primer; R reverse primer; P probe; FAM 6-carboxyfluorescein; TAMRA 6-carboxytetramethylrhodamine; BHQ1 black hole quencher 1.
