# Supplementary material for: Bacterial Exposures and Associations with Atopy and Asthma in Children
Source: PLoS One. 2015 Jun 29;10(6):e0131594. doi: 10.1371/journal.pone.0131594 (PMC4488145; doi:10.1371/journal.pone.0131594)
Supplement: S3 Table — (DOC) [file pone.0131594.s003.doc]

S3 Table. Levels of microbes detected using qPCR (cells/m2 dust) in the three exposure strata stratified by health outcomes.

|  |  | FARM CHILDREN | | | EXPOSED NON-FARM CHILDREN | | | NON-EXPOSED NON-FARM CHILDREN | | |
| --- | --- | --- | --- | --- | --- | --- | --- | --- | --- | --- |
|  |  | ASTHMA | NO ASTHMA BUT ATOPY | NO ASTHMA OR ATOPY | ASTHMA | NO ASTHMA BUT ATOPY | NO ASTHMA OR ATOPY | ASTHMA | NO ASTHMA BUT ATOPY | NO ASTHMA OR ATOPY |
|  |  |  |  |  |  |  |  |  |  |  |
| *Mycobacterium* spp. | Median | 1252815 | 872049 | 868964 | 738266 | 583315 | 876830 | 326199 | 360554 | 690282 |
|  | (min-max) | (33387-1.27x10E7) | (63678 -9157633) | (59851-2.14x10E7) | (28007- 4837331) | (8919 -5347428) | (26067–6195071) | (11049- 5833868) | (20305-1639335) | (38719-4705713) |
|  |  |  |  |  |  |  |  |  |  |  |
| *Bifidobacteriaceae*  spp. | Median | 7853091 | 6882662 | 5168782 | 4933515 | 2054042 | 4330676 | 4337218 | 3317576 | 6571929 |
|  | (min-max) | (64449- 1.78x10E8) | (37103-7.38x10E7) | (220082- 2.55x10E8) | (40180- 4.79x10E7) | (62318-4.22x10E7) | (263633-7.94x10E7) | (236274-2.07x10E8) | (83188- 7.49x10E7) | (57871- 4.76x10E8) |
|  |  |  |  |  |  |  |  |  |  |  |
| *Clostridium*  cluster I | Median | 264510 | 131510 | 106416 | 32028 | 30665 | 32147 | 31438 | 14547 | 73044 |
|  | (min-max) | (255- 1.01x10E8) | (647- 2780997) | (2586-1.73x10E7) | (510- 343781) | (189- 568671) | (1336- 314634) | (638 -257087) | (587- 214262) | (223- 2.98x10E8) |
|  |  |  |  |  |  |  |  |  |  |  |
| *Clostridium* cluster XI | Median | 277263 | 172193 | 271469 | 54883 | 24904 | 54771 | 54362 | 35117 | 90370 |
|  | (min-max) | (621-1.67x10E7) | (1763- 5578865) | (13391- 2.82x10E7) | (4194- 467188) | (69-793189) | (4026- 958813) | (1563- 539362) | (3283-497344) | (1113-1728749) |
|  |  |  |  |  |  |  |  |  |  |  |
