# Supplementary material for: Bacterial Exposures and Associations with Atopy and Asthma in Children
Source: PLoS One. 2015 Jun 29;10(6):e0131594. doi: 10.1371/journal.pone.0131594 (PMC4488145; doi:10.1371/journal.pone.0131594)
Supplement: S1 Text — (DOC) [file pone.0131594.s004.doc]

# Materials & Methods

## Dust samples

Mattress dust samples were collected in nylon socks by vacuuming the entire mattress surface [1]. The dust samples were stored at -80 °C.

## DGGE analyses

An approximately 200 bp fragment of the 16S rRNA gene was amplified using universal bacterial primers [2]. PCR-reactions were carried out in 0.2 ml PCR tubes in a total volume of 50 µl. The reaction mixture contained 400 nM primers (forward primer: 5´-CCT ACG GGA GGC AGC AG-3´ containing a GC clamp: CGC CCG CCG CGC GCG GCG GGC GGG GCG GGG GCA CGG GGG G and reverse primer: 5´-ATT ACC GCG GCT GCT GG-3´), 250 μM dNTPs, Biotools PCR buffer (B&M Labs, Spain), 5% DMSO (Sigma-Aldrich, Germany), 0.5 mM Betain (Sigma-Aldrich, Germany), 0.5 mg/ml BSA (New England BioLabs), and 2.5U of Ultratools DNA polymerase (B&M Labs, Spain).

PCR was performed in a Tgradient PCR cycler (Whatman Biometra, Germany). Cycling parameters were 95 °C for 5 min followed by 35 thermal cycles of 30 sec 95°C, 30 sec 58°C and 30 sec 72°C. A final extension step of 5 min at 72°C was performed.

PCR products were separated using 8 % (v/v) polyacrylamide gel (acrylamide:bisacrylamide 37.5:1, Sigma-Aldrich, Germany). The denaturing gradient (30-70 %) was formed with formamide and urea; 100 % denaturant consisting of 40 % (v/v) formamide and 7 M urea. Electrophoresis was performed in 0.5 x TAE buffer (Amresco, USA) at 60 °C and 175 V 10 min and then 75 V 16h using Dcode Universal Mutation detection system (BioRad, USA).

The DGGE band patterns were visualised by SYBR Gold staining and Dark Reader transilluminator (Claire chemical research). Digital images were captured with Canon PowerShot G9. The images were loaded into the Bionumerics (version 4.61) software and analysed.

## Cloning and sequencing

Selected bands were cut out from the gel, re-amplified and cloned. Analysed PCR-products were re-run on the DGGE gel to confirm correct positioning of the bands in the gel. PCR products were purified using GFX PCR, DNA and gel band purification kit (GE Healthcare, Sweden). 1 µl of purified PCR product was ligated into the pGEM-T vector system (Promega, USA). Ligation products were transformed into *Escherichia coli* competent cells. The transformants were plated on LB agar containing ampicilline, X-cal and IPTG. Plates were incubated at 37 °C 16h.

Plasmid DNA was extracted using the QIAprep spin miniprep kit (Qiagen, Germany). At least 10 plasmids per band were sequenced. The inserts were sequenced with ABI 310 Genetic Analyzer (Applied Biosystems, USA). Clone sequences were aligned against the 16S ribosomal RNA sequences at GenBank DNA database using the Basic Local Alignment Search Tool (BLAST). Genus or family names of highly similar database entries were provided where sequence similarity to clone sequences was ≥ 99%. A suggestion for the taxonomic allocation of the DGGE band was provided where the recovered clone sequences pointed clearly towards a distinct taxonomic group.

Detailed criteria for band selection for sequencing were described in the Methods section in the main manuscript. In essence, bands with significant associations to doctor diagnosis of asthma and/or atopy and/or doctor diagnosis of atopic eczema were selected. An exception was band 15 that did show significant associations with atopy, but was not sequenced due to a miscommunication in the laboratory process. Band 10 was sequenced instead, but since this band is of no particular interest for this study (no associations to health or exposure variables) the sequencing results are not shown.

## qPCR

Literature search was conducted identifying suitable qPCR assays. All of the selected assays – except *Mycobacterium* spp. – had to be optimized for the mattress dust samples. Primers, probes and optimized running conditions are presented in Table S1. All qPCR assays based on previously published primer/probe systems and the specificity of these assays has been tested and described. For the *Mycobacterium* spp. assay [3] the specificity was validated through BLAST searching against the GenBank database (http://www.ncbi.nlm.nih.gov/blast) and Probe Match algorithm of the Ribosomal Database project (RDP; http:// <http://rdp.cme.msu.edu/probematch/search.jsp>). The sensitivity and specificity was tested against 26 *Mycobacterium* strains and 10 closely related non-mycobacteria strains. The primer pair and probe were shown to be specific for mycobacterial sequences. The assays for *Clostridium* spp. clusters I and XI were developed and tested in the work by Song et al. [4]. The authors described extensive cross-specificity checks on the RDP database using CHECK_PROBE utility and comparison to 16S rDNA databases using BLAST that led to the final selection of the qPCR assays. The specificity was tested in experimental conditions using 54 Clostridium strains and 22 strains of closely related bacteria. The *Clostridium* cluster I qPCR assay was found to be specific for most of the species allocated to cluster I and three species of cluster II [5]. The *Clostridum* cluster XI qPCR assay was found to be specific for part of *Clostridum* cluster XI species [4]. Development and specificity testing of the *Bifidobacteriaceae* spp. assay has been reported by authors [6]. Specificity of the primer/probe set was tested in silico using BLAST, and under experimental conditions using 11 *Bifidobacterium* spp. and mixtures of 21 other, non-target strains. The authors report the assay to be highly specific for *Bifidobacterium* spp. Our own testing with Primer-Blast and Probe Match algorithm at RDP revealed possible amplification of closely related genera within the family *Bifidobacteriaceae*, eg. *Gardnerella* spp., which is why this assay is referred to here as *Bifidobacteriacea* spp..

The total volume of the qPCR reaction was 25 µl. *Mycobacterium* spp. qPCR was performed as described by Torvinen et al. [3]. *Bifidobacteriaceae* spp. and both *Clostridium* assays consisted of 12 µl of DyNAmo™ Probe qPCR Kit mastermix (Finnzymes, Finland), 2.5 µl of BSA (2 mg/ml) and HyClone® HyPure™ nuclease free water (HyClone Laboratories Inc., USA). *Clostridium* cluster I contains 150 nM each oligonucleotide, *Clostridium* cluster XI 200 nM forward and reverse primers and 100 nM probe. *Bifidobacteriacea* spp. primer concentrations are 100 nM and probe 200 nM. Finally, 2 µl of template DNA was added in each well. All qPCR assays were performed using the Rotor-Gene 3000 PCR instrument (Corbett Research, Australia). The internal control, *Geotrichum candidum*, was detected with ABI Prism 7000 (Applied Biosystems, USA).

Numbers of detected cell equivalents in the samples were calculated using relative quantification as described earlier [7]. Shortly, to calculate the microbial cell equivalents in the dust sample, target Ct values were normalised using the internal reference (*G. candidum*). Ct value and the average of the standard curve were used as the control sample. Standard curves were produced using from one to three different pure strains of the detected microbes per standard curve. DNA was isolated from 100 μl of the bacterial suspensions with a known concentration, and tenfold serial dilutions of the isolated DNA were analysed in triplicate using qPCR assays. The amplification efficiency (Ae) of the primer/probe set was calculated using the formula: Ae=10^(−1/slope)^, in which the slope was calculated by regression analysis of the obtained Ct values versus calculated log number of cells in the serial dilutions.

# References

1. Genuneit J, Buchele G, Waser M, et al. The GABRIEL advanced surveys: Study design, participation and evaluation of bias. Paediatr Perinat Epidemiol. 2011;25(5):436-447.

2. Muyzer G, de Waal EC, Uitterlinden AG. Profiling of complex microbial populations by denaturing gradient gel electrophoresis analysis of polymerase chain reaction-amplified genes coding for 16S rRNA. Appl Environ Microbiol. 1993;59(3):695-700.

3. Torvinen E, Torkko P, Rintala AN. Real-time PCR detection of environmental mycobacteria in house dust. J Microbiol Methods. 2010;82(1):78-84.

4. Song Y, Liu C, Finegold SM. Real-time PCR quantitation of clostridia in feces of autistic children. Appl Environ Microbiol. 2004;70(11):6459-6465.

5 .

6. Penders J, Vink C, Driessen C, Thijs C, Stobberingh EE. Quantification of Bifidobacterium spp., Escherichia coli and Clostridium difficile in faecal samples of breast-fed and formula-fed infants by real-time PCR*. FEMS Microbial Let*t; 2005: 243(1):141-147.

7. Haughland RA, Varma M, Wymer LJ, Vesper SJ. Quantitative PCR analysis of selected Aspergillus, Penicillium and Paecilomyces species. Syst. Appl. Microbiol., 2004, 27(2), 198–210.
